# Non–Exercise-Based Interventions to Support Healthy Aging in Older Adults: A Systematic Review and Meta-Analysis of Randomized Controlled Trials

**DOI:** 10.1093/geront/gnae156

**Published:** 2024-10-30

**Authors:** Wei Qi Koh, Nutyathun Wora, Natasha Wing Laam Liong, Kristiana Ludlow, Nancy A Pachana, Jacki Liddle

**Affiliations:** School of Health and Rehabilitation Sciences, The University of Queensland, Brisbane, Queensland, Australia; School of Psychology, The University of Queensland, Brisbane, Queensland, Australia; Faculty of Psychology, Chulalongkorn University, Bangkok, Thailand; School of Health and Rehabilitation Sciences, The University of Queensland, Brisbane, Queensland, Australia; Centre for Health Services Research, The University of Queensland, Brisbane, Queensland, Australia; School of Psychology, The University of Queensland, Brisbane, Queensland, Australia; School of Health and Rehabilitation Sciences, The University of Queensland, Brisbane, Queensland, Australia; Department of Occupational Therapy, Princess Alexandra Hospital, Brisbane, Queensland, Australia

**Keywords:** Active aging, Aging well, Healthy aging, Health promotion, Older adults

## Abstract

**Background and Objectives:**

Healthy aging is a multidimensional construct, where various factors play a role in influencing well-being in older age. Many studies heavily emphasize the importance of physical activity, negating other aspects such as psychosocial or cognitive influences. This review aimed to evaluate the effectiveness of non-exercise-based interventions on the quality of life (QoL) and life satisfaction of community-dwelling, healthy older adults.

**Research Design and Methods:**

A systematic review and meta-analysis of randomized controlled trials was conducted. Four electronic databases were searched from inception. Three independent reviewers screened the articles and appraised the risk of bias. Data relating to study characteristics, interventions, and outcomes were extracted. The mean difference and standardized mean difference with 95% confidence intervals (CI) were synthesized to pool effect sizes. Outcomes that could not be included in the meta-analysis were synthesized narratively.

**Results:**

Thirty-six articles from 35 randomized controlled trials, involving 6,127 older adults, were included. Most were multicomponent interventions and involved supporting lifelong learning. Most participants were females (70.2%). Pooled analyses revealed small but statistically significant effects on overall QoL (standardized mean difference [SMD] = 0.26, CI: 0.00 to 0.53), and QoL subscales relating to mental health and psychological well-being (SMD = 0.26, CI: 0.12 to 0.41) and environment (SMD = 0.31, CI: 0.03 to 0.58). Effects on social health and functioning, and role functioning related to physical health were marginal. There were no improvements in other subscales. Results revealed nonstatistically significant improvements in life satisfaction.

**Discussion and Implications:**

Multicomponent non-exercise-based interventions that support lifelong learning in older adults can support healthy aging, particularly in improving overall QoL, and mental health, social health, and environment QoL subscales.

The World Health Organization (2024) estimates that 1 billion of the world’s population was aged 60 years and older in 2019; this figure is expected to reach 2.1 billion by 2050. Accordingly, the need to support older people to age well and to lead healthy, active, and independent lives for as long as possible has become ever more pertinent. In the last decade, there has been a proliferation of interventions to support older adults to age actively, particularly interventions to support physical activities, chronic disease self-management, healthy eating, and social functioning (Owusu-Addo et al., 2021). Physical activity and exercise play a pivotal role in supporting older adults to live well in the community. In a meta-analysis of 30 randomized controlled trials (RCTs) and quasi-randomized trials, Kumar et al. (2016) found that exercise-based interventions like Taichi, yoga, balance, strength, and resistance training, demonstrated small-to-moderate effects on reducing fear of falling amongst community-dwelling older people. These resonated with findings from similar and more recent systematic reviews by Sadaqa et al. (2023) and Di Lorito et al. (2021). A growing evidence base supports the wider benefits of physical activity on nonphysical domains such as cognition, for instance, working memory, as demonstrated through synthesized findings from 36 RCTs (Northey et al., 2018). The importance of physical activity is well recognized, and evident through community services that focus primarily on addressing or supporting the physical needs of older adults (Brett et al., 2019; Turcotte et al., 2015). However, there is an overemphasis on physical activity in healthy aging research, negating other factors contributing to healthy aging, such as psychosocial aspects (Malkowski et al., 2023). While exercise-based trials could focus on evaluating nonphysical outcome measures, it is also necessary to evaluate the role of non-exercise-based pillars of healthy aging in supporting older adults’ activity participation and quality of life (QoL).

In addition to physical function and avoiding disease and disability, being actively engaged in life is an important correlate of healthy aging (Annele et al., 2019; Dogra et al., 2022; Fernandez-Ballesteros, 2019; Kim & Park, 2017). Healthy aging refers to “the process of developing and maintaining the functional ability that enables wellbeing in older age” (World Health Organization, 2020). It encompasses not only physical aspects but also the psychological and social aspects of health. Furthermore, being engaged in daily routines and a range of meaningful activities have also been highlighted as an important aspect of aging well. This has been well illustrated through McKinsey Health Institute’s (2023) survey of over 21,000 older people from over 21 countries, which revealed that aging well entailed having a sense of purpose, managing stress, enjoying meaningful connections with others, and preserving independence. In qualitative studies involving Australian older adults (Buys and Miller, 2006) and older people from Thailand (Wongsala et al., 2021), aging well has been described as having active participation in social interactions, daily routines, personal development, and having adaptive mindsets, and engaging in altruistic work. These findings were echoed in a study involving Indigenous older people, who reported viewing aging well as achieving holistic health and well-being by staying physically, cognitively, and purposefully engaged (Quigley et al., 2022). These findings highlight the importance of a holistic approach to healthy aging and need to focus our understanding on healthy aging beyond physical health determinants. The aim of this study was to evaluate the effectiveness of non-exercise-based interventions on healthy ageing, specifically in relation to life satisfaction and QoL, as these are more meaningful parameters for older adults than specific component-based outcomes, such as cognitive gains. QoL is a meaningful outcome measure for older adults because it is a critical component of public health (Rejeski & Mihalko, 2001). A number of international action plans on aging have also emphasized the importance of measuring QoL as a person-level outcome (Beard et al., 2016; Network, 2016; Van Leeuwen et al., 2019).

## Method

The review protocol was registered with PROSPERO (registration number: CRD42023481588), following the PRISMA guidelines. Deviations were reported in PROSPERO and justified below. The Population, Intervention, Comparison, Outcome, and Study Design (PICOS) framework was used to structure this review. PubMed, Medline (EBSCO), PsycINFO (EBSC), and CINAHL (EBSCO) were searched in October 2023 with no date limits applied. Supplementary Appendix 1 provides a detailed example of our search strategy. The reference lists of included articles were screened for potentially relevant articles.

### Selection Criteria

The eligibility criteria (Supplementary Appendix 2) were as follows: (1) RCTs on non-exercise-based interventions that were focused on supporting any nonphysical aspects of aging, such as psychological, social, and/or cognitive well-being, (2) published in English and included (3) older adults aged 55 and older, are living in the community, independent in self-care, and did not have any physical, cognitive, or psychological impairments and disabilities, such as stroke, depression, or dementia that limit their functional independence to ensure that interventions were targeted at healthy aging rather than rehabilitation or disability self-management, and (4) measured QoL and life satisfaction assessed using standardized outcome measures. In deviation from our protocol, we excluded our secondary outcome of interest to investigate participation in daily activities. This was due to a large range of potential outcomes of interest ranging from self-care, instrumental activities, social and a wide range of leisure activities. Authors of journal articles were contacted for more information to clarify inclusion criteria as needed. The exclusion criteria were (1) non-RCTs, such as quasi-experimental studies, (2) adults aged below 55, not living in independently, and (3) interventions focused on rehabilitation or disease self-management, or contained any exercise components.

Search results were imported into Covidence, a reference management software, and duplicates were removed. The titles and abstracts of articles were independently screened by W. Q. Koh and N. Wora, and full-text screening was conducted independently by W. Q. Koh and N. W. L. Liong. Conflicts were resolved through discussions.

### Data Extraction

Data were extracted on a standardized data extraction form on Microsoft Excel. They include: (i) study characteristics (author, country, setting), (ii) participants’ characteristics (age, gender, marital status, living arrangement, sample size), (iii) intervention and control characteristics (nature of interventions, frequency), (iv) outcome measures, and (v) life satisfaction and/or quality of life outcomes. The healthy aging domains that interventions addressed were categorized based on determinants of healthy aging outlined in a systematic review (Abud et al., 2022). They include two physical domains (diet and physical activity), four mental and cognitive domains (self-awareness, outlook/attitude, lifelong learning, and faith), three social domains (social support, financial security, and community engagement), and one domain relating to independence and self-reliance.

### Risk of Bias Assessment

Three reviewers independently assessed the risk of bias using the Cochrane Risk of Bias 2 (RoB-2) tool for assessing risk of bias in randomized trials (Sterne et al., 2019). This included assessing biases that arose from the randomization process, outcome completeness and measurement, and reporting. W. Q. Koh assessed all articles, and N. Wora and N. W. L. Liong each assessed 50%. All reviewers piloted the assessment on two articles using the RoB-2 guidance and met to discuss and agree on discrepancies in appraisal approaches before proceeding to appraise the remaining articles. Conflicts were resolved through discussions.

### Data Synthesis

Outcome data were imported into Statistical Packages for Social Sciences (SPSS) version 29.0 for meta-analysis. The effect sizes of continuous outcome data were calculated by using the standardized mean difference (SMD) or mean difference (MD). A random-effects model based on the generic inverse method was applied, and heterogeneity between studies was measured using the Chi-square test (*p* < .10 and *I*^2^ > 40%). Studies with multiple arms resulted in the findings from relevant groups being combined to create a pair-wise comparison. Outliers were identified using the leave-one-out method. To explore the observed heterogeneity, we performed additional subgroup analyses where possible for additional variables created: (i) intervention duration (4–6 weeks, >6–12 weeks, >12–26 weeks, >26 weeks), (ii) mean age (≤75 years or >75 years), and (iii) intervention facilitation, which indicated whether interventions were delivered by nonhealth professionals, health professionals, or a combination of both. Subgroup analysis was not viable for intervention types as some studies adopted a combination of different intervention components and for participant characteristics that were not reported in all studies (e.g., living arrangements and marital status). Outcomes that could not be included in the meta-analysis (e.g., were not presented in an appropriate format) were synthesized narratively, where descriptive textual information was extracted, organized into groups, and presented (Popay et al., 2006).

## Results

**Figure 1.**
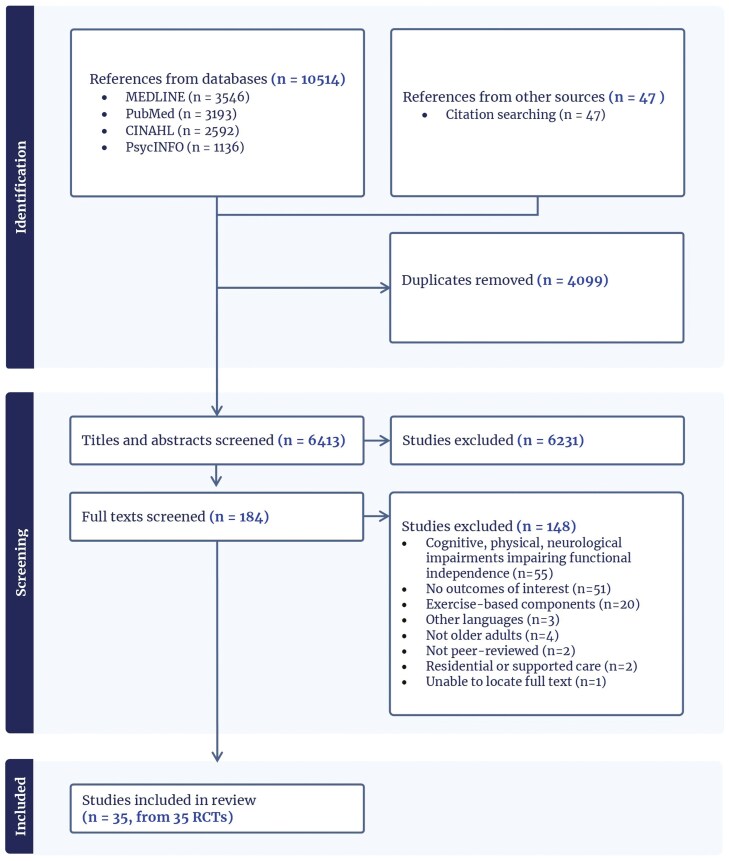
PRISMA flow diagram. PRISMA = xxx.

In all, 10,467 articles were retrieved from the database search, and 47 articles were retrieved through reference list searches. After deduplication, 6,385 articles were screened for eligibility, following which the full texts of 165 articles were screened. Thirty-five articles from 31 studies that met the eligibility criteria were included (Figure 1, PRISMA flow diagram).

### Risk of Bias Appraisal

**Figure 2.**
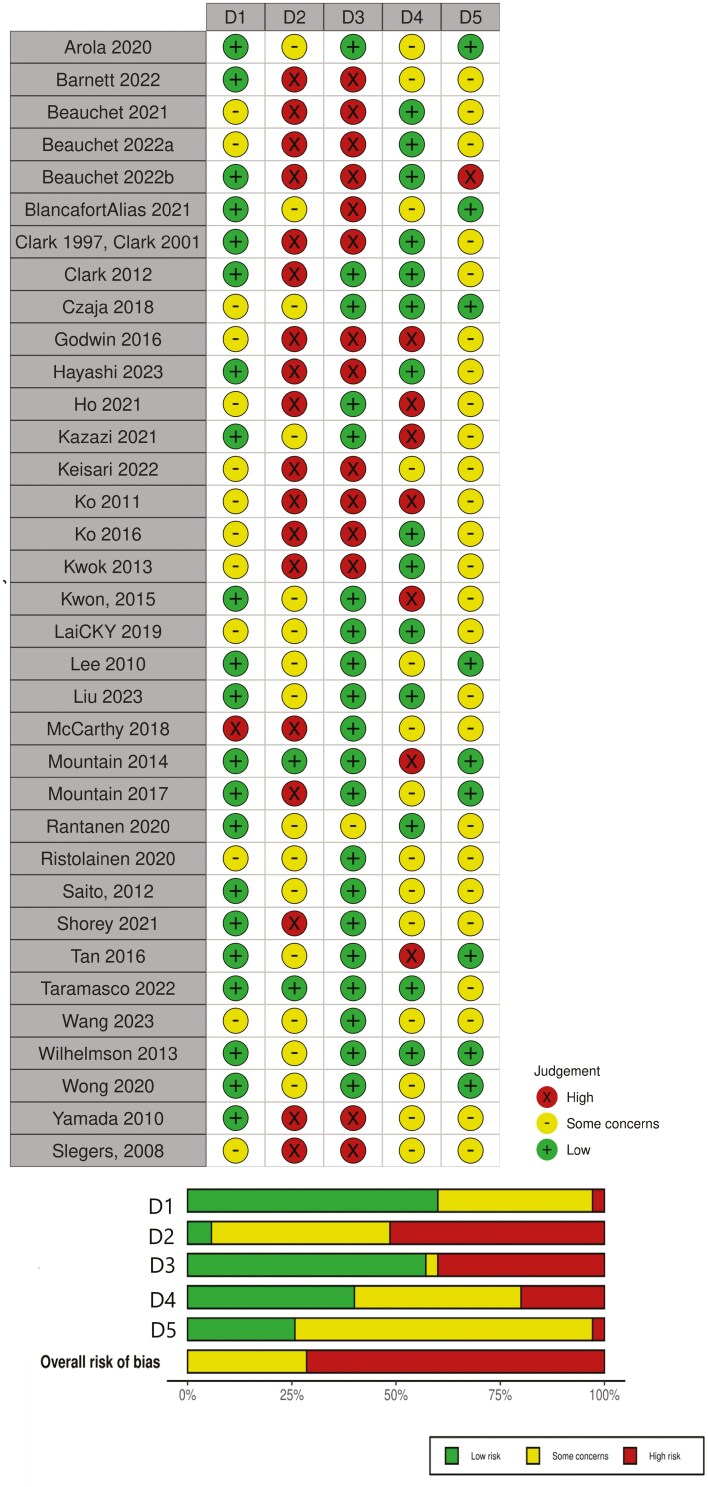
Risk of bias.

The risk of bias is presented in Figure 2 (McGuinness & Higgins, 2020). Most studies (*n* = 21, 60%) demonstrated a low risk of bias in domain one, which relates to bias arising from the randomization process. In domain 2 which assesses bias relating to deviation from intended intervention, most (*n* = 27, 77.1%) did not provide information on deviations and did not perform intention-to-treat analyses (*n* = 19, 54.3%). Accordingly, most had a moderate to high risk of bias in this domain (*n* = 33, 94.3%). Regarding missing outcome data (domain three), 15 studies (48.6%) did not provide information on missing data management. For bias relating to the measurement of outcome data (domain 4), all used appropriate outcome measures. Biases arose from having nonblinded outcome assessors in 11 studies (31.4%). Eight studies (22.9%) did not provide information on outcome assessor blinding. In domain 5, most studies were at moderate or high risk of bias in relation to result selection bias due to a lack of prespecified analysis intentions through preregistration or protocols (*n* = 27, 77.1%).

### Study Characteristics

Different randomized controlled study designs were used, the most common being two-arm (parallel) trials, cross-over studies, and multiple-arm trials. Studies were conducted in 14 countries across America, Europe, and Asia which included Canada (*n* = 2), USA (*n* = 4), Chile (*n* = 1), the UK (*n* = 2), Finland (*n* = 2), Sweden (*n* = 2), Spain (*n* = 1), Korea (*n* = 4), Japan (*n* = 3), Hong Kong (*n* = 3), Singapore (*n* = 2), Taiwan (*n* = 2), Iran (*n* = 1), and Israel (*n* = 1). One was a cross-national study between Japan and Canada (*n* = 1). A summary of the study characteristics can be found in Supplementary Appendix 3.

### Participants’ Characteristics

In all, 6,127 older adults were included in the review. The number of participants in each study ranged from 20 to 460. From 35 studies that reported mean age or age ranges, participants’ ages ranged from 64 to 85.5 years (64.4 to 97 years in control group, 65.4 to 94 years in intervention group). Females constituted 70.2% of the sample in contrast to males (29.8%). In 20 studies (71.4%) that reported on living arrangements, 63.1% (*n* = 4,033) lived alone. Fifteen studies (42.9%) that reported on participants’ marital status revealed that 46.0% (*n* = 861) were married or cohabiting, and 54.0% (*n* = 1,011) were single, divorced, widowed, or separated.

### Intervention Characteristics

A summary of the intervention characteristics can be found in Supplementary Appendix 3, and Supplementary Appendix 4 shows categorization of interventions into healthy aging domains. Most trials (*n* = 25, 71.4%) supported lifelong learning, which entailed education or information provision (*n* = 16); problem solving and/or goal setting (*n* = 7); engaging in a range of leisure activities (*n* = 13); and computer training (*n* = 1). Information provision or education involved a range of topics relating to healthy aging, well-being, occupational participation, and/or aging-related challenges. Leisure-based interventions included creative arts (*n* = 7); guided museum tours (*n* = 3); creating and/or sharing life stories (*n* = 4, 11.4%) through drama therapy, life storybook, and life reviews; laughter therapy (*n* = 1); pet care (*n* = 1); and music therapy (*n* = 1). Two studies delivered leisure interventions via videoconferencing during coronavirus disease 2019 (COVID-19) (Beauchet, Matskiv, et al., 2022; Liu et al., 2023). Three studies (*n* = 3) focused on targeting specific cognitive components and mindfulness, and one (*n* = 1) provided training on smartphone and app use.

Ten studies (*n* = 10) aimed to increase participants’ self-awareness (*n* = 8) and attitudes (*n* = 2) by facilitating reflection or introspection about their daily lives, habits, personal experiences, meaningful aspects, and/or challenges, or to develop self-acceptance and an integrative view of the self. In the social domain, 13 trials (*n* = 13) reported interventions that provided or facilitated social support, three of which were digital interventions delivered via videoconferencing or telephone. Thirteen studies (*n* = 13) supported community engagement through community-based interventions, such as museums or local neighborhoods (*n* = 8) and/or connecting older adults with local resources (*n* = 7). Seven trials (*n* = 7) as reported in eight studies aimed to facilitate independence by supporting self-management in different aspects of life, including social participation (Blancafort et al., 2021), maintaining overall wellness (Kwon, 2015; Wilhelmson & Eklund, 2013), promoting health through occupational engagement (Clark et al., 1997, 2001, 2012; Mountain et al., 2017; Yamada et al., 2010), and increasing environmental safety through ambient assisted living technology (Taramasco et al., 2022). Only one study focused on supporting older adults’ dietary self-management (Barnett & Zeng, 2022).

In terms of intervention delivery, approximately one-third (*n* = 12) were delivered by health or social care professionals (Arola et al., 2020; Blancafort Alias et al., 2021; Clark et al., 1997, 2001, 2012; Godwin et al., 2016; Ko & Youn, 2011; Kwon, 2015; Mountain et al., 2017; Rantanen et al., 2020; Wilhelmson & Eklund, 2013; Wong & Wong, 2020; Yamada et al., 2010). These included occupational therapists, physiotherapists, nurses, social workers, general practitioners, care staff, and a counselor. In eight studies, facilitators were nonhealth professionals—arts and culture facilitators, professional artists, volunteers, and computer instructors (Beauchet et al., 2021, Beauchet, Cooper-Brown, et al., 2022, Beauchet, Matskiv, et al., 2022; Hayashi et al., 2023; Lai et al., 2019; Mountain et al., 2014; Shorey et al., 2021; Slegers et al., 2008). A combination of health and nonhealth professionals delivered interventions in five studies (*n* = 5; Barnett & Zeng, 2022; Ho et al., 2021; Keisari et al., 2022; McCarthy et al., 2018; Ristolainen et al., 2020).

### Control Conditions

Control participants in most studies received no intervention or usual care (*n* = 22, 62.9%) or were on waitlist control groups (*n* = 5, 14.3%). In five trials (*n* = 5, 14.3%) participants received printed or verbal health information or education. Social activities and craft groups were used as control conditions in four studies (Clark et al., 1997, 2001, 2012; Lai et al., 2019; Yamada et al., 2010). One trial reported providing a range of content and information for participants in the control group (Liu et al., 2023), and one reported providing computer training for one control group (Slegers et al., 2008).

### Intervention Format and Duration

Most interventions were primarily conducted in group settings (*n* = 22). Nine studies (*n* = 9) delivered individual interventions (*n* = 9), and three (*n* = 3) combined individual and group intervention formats. Sixteen studies (*n* = 16) were conducted over a 4 to 8-week period, and 10 studies (*n* = 10) were conducted over a 12–18-week period. Six studies (*n* = 6) reported an overall intervention duration between 6 months and 1 year. This information was not explicit in one study (Slegers et al., 2008), which took place over 4 months, and one study (Shorey et al., 2021) did not provide this information.

Most interventions (*n* = 19) were conducted weekly, of which three (*n* = 3) provided additional interventions (Clark et al., 1997, 2001; Shorey et al., 2021; Wong & Wong, 2020). Four studies (*n* = 4) provided interventions twice weekly, and one (*n* = 1) did so 5 days per week. Others conducted fortnightly (Saito et al., 2012), bimonthly (Yamada et al., 2010), full-time/self-directed interventions (Czaja et al., 2018; Ko et al., 2016), interventions with a range of frequencies (*n* = 1; Slegers et al., 2008) or did not provide explicit information (*n* = 5) on intervention frequency (Godwin et al., 2016; Rantanen et al., 2020; Ristolainen et al., 2020; Shorey et al., 2021; Taramasco et al., 2022). Eleven studies (*n* = 11) reported intervention lengths between 1.5 and 2 hr per session, and nine (*n* = 9) reported length of interventions being between 30 and 60 min. Two studies (*n* = 2) provided interventions lasting between 2.5 and 3 hr (Barnett & Zeng, 2022; Ristolainen et al., 2020).

### Meta-Analysis

Twenty-nine papers from 28 RCTs were included in the meta-analysis. Key forest plots on overall life satisfaction and QoL are presented in the figures below, and those of other QoL subcomponents can be found in Supplementary Appendix 5.

#### Life satisfaction

**Figure 3.**
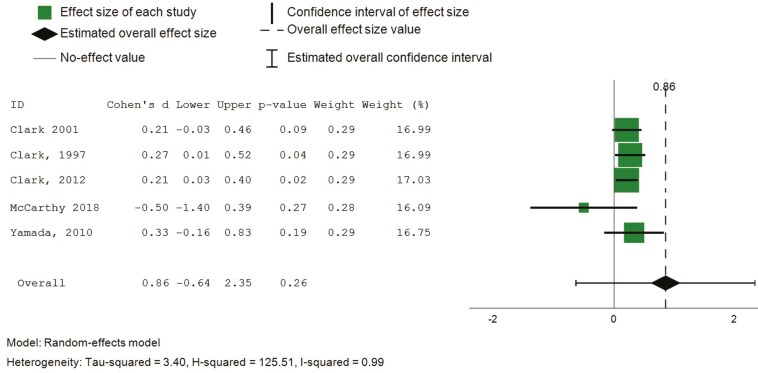
Forest plot—intervention effects (life satisfaction).

Six articles from seven randomized controlled trials used the Life Satisfaction Index (Clark et al., 1997, 2001, 2012; McCarthy et al., 2018; Yamada et al., 2010), and one used the Satisfaction with Life Scale (Keisari et al., 2022). Pooled findings showed a large but not statistically significant effect (6 studies, 621 participants, SMD 0.86, 95% confidence intervals [CI]: −0.64 to 2.35). Upon removal of an outlier (Keisari et al., 2021), the overall pooled estimate revealed a small but statistically significant effect (Figure 3, SMD 0.22, CI: 0.11 to 0.34, *I*^2^ = 0%).

#### Quality of life

Twenty-three (*n* = 23) studies reported on QoL outcomes, using the EuroQol 5-dimension (EQ5D), RAND Short Form Health Survey 36 (SF-36), the Short Form Health Survey 12 (SF-12), the Short Form Health Survey (SF-8), World Health Organization Quality of Life Scale (WHOQoL-BREF), and the Office of National Statistics (ONS) Wellbeing Measure. Some studies reported overall QoL scores, and most studies reported subscale scores, which included (1) mental health or psychological well-being, (2) physical health, (4) general health, (5) social health or functioning, (6) participation in roles (e.g., in work and daily activities) because of physical and mental limitations, and (7) environmental influences on QoL.

##### Overall QoL scores

**Figure 4.**
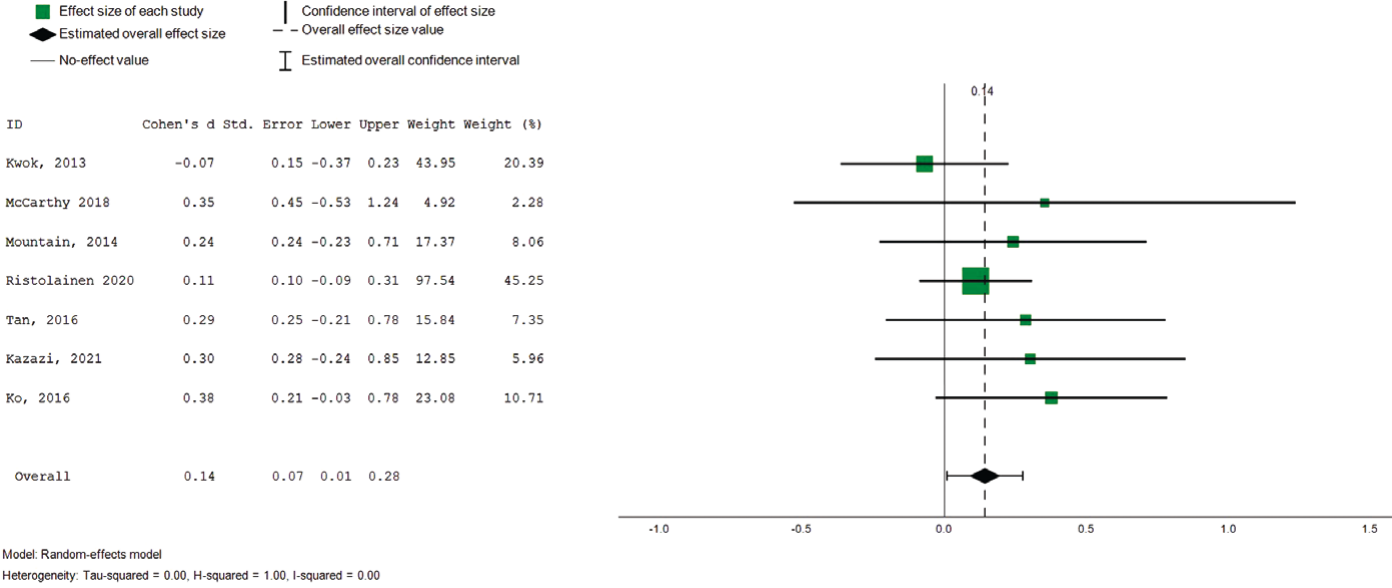
Forest plot—intervention effects (overall QoL).

**Figure 5.**
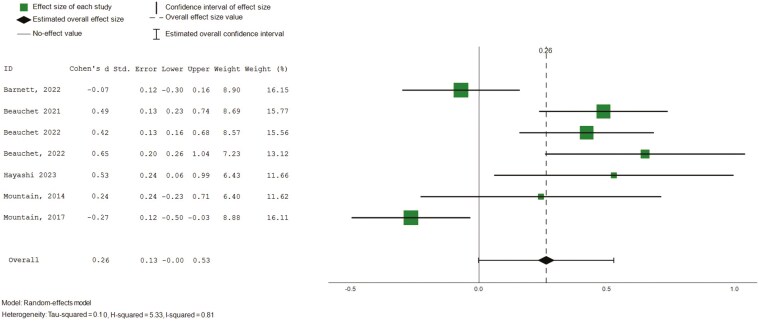
Forest plot—intervention effects (global self-rating on health). Std. = xxx.

Overall QoL was reported as (i) global QoL scores in seven studies (Kazazi et al., 2021; Ko et al., 2016; Kwok et al., 2013; McCarthy et al., 2018; Mountain et al., 2014; Ristolainen et al., 2020; Tan et al., 2016) and reported using the (ii) visual analogue subscale (VAS) scores on the EQ-5D (*n* = 7), a vertical scale that takes value between 0 and 100 on which individuals provide a global self-rating of their health (Barnett & Zeng, 2022; Beauchet et al., 2021; Beauchet, Cooper-Brown, et al., 2022; Beauchet, Matskiv, et al., 2022; Mountain et al., 2014, 2017). Pooled findings revealed small but statistically significant effects on global QoL scores (Figure 4, 7 studies, 420 participants, SMD 0.14, CI: 0.03 to 0.58, *I*^2^ = 0%). Subgroup analysis was only possible by age group and no subgroup effect was observed. On the VAS subscale of the EQ-5D, pooled analysis showed statistically significant small effect sizes with substantial heterogeneity (Figure 5, 7 studies, 784 participants, SMD 0.26, CI: 0.00 to 0.53, *I*^2^ = 81%). Subgroup analysis revealed statistically significant moderate effects for interventions facilitated by nonhealth professionals (5 studies, 489 participants, SMD 0.47, CI = 0.32 to 0.62, *I*^2^ = 0%). No subgroup effects were observed by age groups.

##### Mental and psychological health

**Figure 6.**
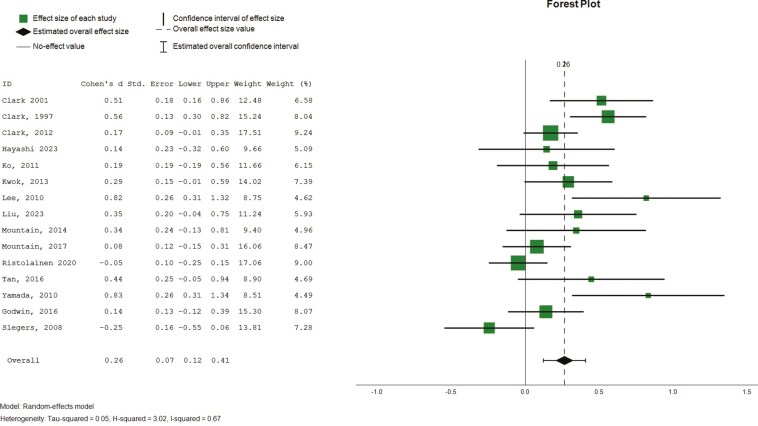
Forest plot—intervention effects (mental and psychological health). Std. = xxx.

Fifteen studies (*n* = 15) reported the mental health component of the SF-36, and the psychological component of the WHOQoL-BREF were reported in 14 studies (Clark et al., 1997, 2001, 2012; Godwin et al., 2016; Hayashi et al., 2023; Kwok et al., 2013; Lee et al., 2010; Liu et al., 2023; Mountain et al., 2014, 2017; Ristolainen et al., 2020; Slegers et al., 2008; Tan et al., 2016; Yamada et al., 2010). Five studies (Clark et al., 2012; Ko et al., 2016; Kwon, 2015; Mountain et al., 2014, 2017) also reported mental component summary scores based on SF-36. Pooled findings revealed a small but statistically significant effect on mental and psychological well-being (Figure 6, 15 studies, 1,213 participants, SMD 0.26, CI: 0.12 to 0.41, *I*^2^ = 67%). Subgroup effects were observed for intervention duration (4–12 weeks; SMD 0.36, CI = 0.19 to 0.54, *p* = .00; 4–6 months, SMD = 0.05, CI = −0.09 to 0.18, *p* = .49; >6 months, SMD 0.47, CI = 0.15 to 0.20, *p* = .00), interventions facilitated by nonhealth professionals (SMD = 0.06, CI = −0.29 to 0.42, *p* = .73) and health professionals (SMD = 0.30, CI = 0.16 to 0.45, *p* = .00).

##### Physical health and functioning

Seventeen studies reported on physical health and functioning aspects of QoL using the EQ-5D, SF-36, SF-12, WHOQoL, and the WHOQoL-BREF (Beauchet et al., 2021; Beauchet, Cooper-Brown, et al., 2022; Beauchet, Matskiv, et al., 2022; Clark et al., 1997, 2001, 2012; Godwin et al., 2016; Hayashi et al., 2023; Ko & Youn, 2011; Kwok et al., 2013; Lee et al., 2010; Liu et al., 2023; Mountain et al., 2014, 2017; Ristolainen et al., 2020; Slegers et al., 2008; Tan et al., 2016). Five studies (Clark et al., 2012; Ko et al., 2016; Kwon, 2015; Mountain et al., 2014, 2017) reported physical component summary scores based on the SF-36, which combines the physical functioning, physical influences on role functioning, and general health. Results showed marginal and nonstatistically significant intervention effects on physical health and functioning (Supplementary Appendix 5, 17 studies, 1,603 participants, SMD 0.09, CI: −0.11 to 0.30, *I*^2^ = 87%). No subgroup effects were found for intervention duration, age group, and facilitation.

##### Social health and functioning

**Figure 7.**
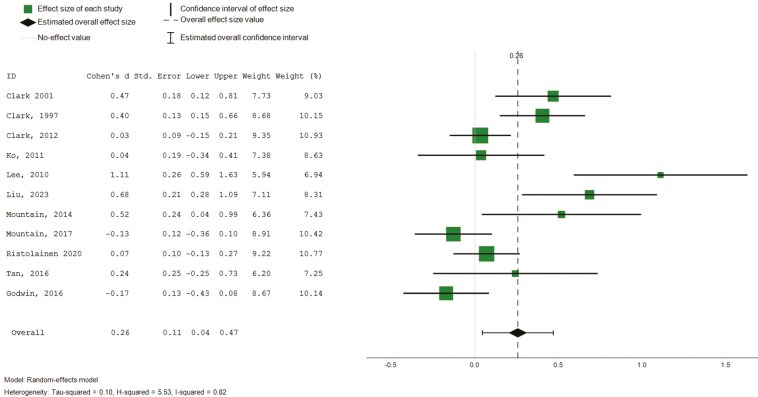
Forest plot—intervention effects (Social health and functioning). Std. = xxx.

Eleven studies (Clark et al., 1997, 2001, 2012; Godwin et al., 2016; Ko & Youn, 2011; Lee et al., 2010; Liu et al., 2023; Mountain et al., 2014, 2017; Ristolainen et al., 2020; Tan et al., 2016) reported on social functioning. Pooled findings demonstrated marginal but statistically significant effects (Figure 7, 11 studies, 1,001 participants, SMD 0.03, CI: 0.04 to 0.47, *I*^2^ = 82%). Subgroup analysis could only be conducted for intervention duration, which did not explain substantial heterogeneity.

##### Role functioning (physical and emotional)

**Figure 8.**
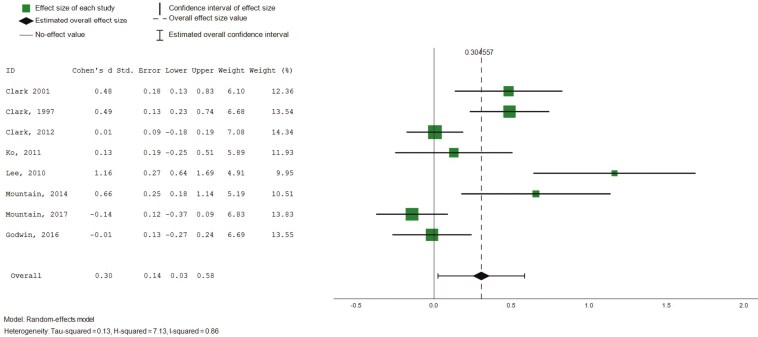
Forest plot—intervention effects (role functioning and physical). Std. = xxx.

Eight studies (*n* = 8) reported on intervention effects relating to emotional and physical influences on role functioning, based on SF-36 (Clark et al., 1997, 2001, 2012; Godwin et al., 2016; Ko & Youn, 2011; Lee et al., 2010; Mountain et al., 2014, 2017). Pooled analysis revealed small and nonstatistically significant effects on the emotional influences of role functioning (8 studies, 734 participants, SMD 0.18, CI: −0.09 to 0.44), and small, statistically significant effects in role functioning relating to physical health (Figure 8, 8 studies, 734 participants, SMD 0.31, CI: 0.03 to 0.58, *I*^2^ = 84%). Subgroup analysis was conducted for intervention duration, which did not explain substantial heterogeneity.

##### Environmental influences

**Figure 9.**
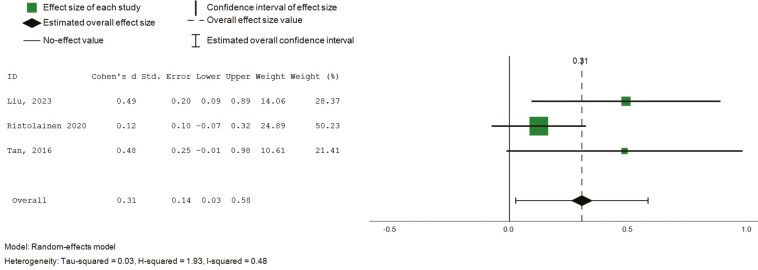
Forest plot—intervention effects (environment). Std. = xxx.

Three studies (*n* = 3) reported on effects on environmental influences on QoL based on the environmental subscale of the WHOQoL tool (Liu et al., 2023; Ristolainen et al., 2020; Tan et al., 2016), with findings demonstrating small but statistically significant effects (Figure 9, 3 studies, 267 participants, SMD 0.31, CI: 0.03 to 0.58). Subgroup analysis could not be conducted.

### Narrative Synthesis

Seven studies (*n* = 7) that did not provide data in a suitable format or did not provide sufficient raw data were narratively summarized. Six studies evaluated life satisfaction, of which three (*n* = 3) reported that life satisfaction measures remained the same or improved in interventions that involved life story and social support programs (Lai et al., 2019; Saito, 2012), however, results were not statistically significant in the study by Arola et al. (2020), which delivered a home-based health promotion intervention. Wong and Wong (2020) and Shorey et al. (2021) delivered health promotion and mindfulness interventions, respectively, and found no significant changes in life satisfaction scores. In a preventative home visit intervention program, Wilhelmson and Eklund (2013) found that life satisfaction scores decreased in both the intervention and control groups, with decreases being more pronounced in the latter.

Four studies evaluated QoL outcomes. Czaja et al. (2018) found that a computer system for older adults led to statistically significant improvements in the overall QoL score of older adults but led to a decrease in the physical functioning subscale. Three studies (*n* = 3) reported that life story, mindfulness, and a self-care intervention program did not have statistically significant effects on the mental health and psychological well-being QoL subscales (Lai et al., 2019; Shorey et al., 2021; Wong & Wong, 2020).

## Discussion

There is a growing imperative for interventions to support older adults to age well, and more knowledge is needed to understand the role of non-exercise interventions. To the best of our knowledge, this is the first review to synthesize evidence on the effectiveness of non-exercise-based interventions to support healthy aging in older adults, specifically in relation to improving life satisfaction and QoL. Pooled results from the meta-analysis demonstrate that they significantly improved overall QoL in older adults, and in the mental health, social health, and environment subscales of QoL. Both the meta-analysis and narrative summary also demonstrate the potential of such interventions to improve life satisfaction in older adults, however, results were mixed. There were no improvements to the physical and emotional role functioning subscales of QoL.

Most included studies had moderate to high risks of biases, especially in relation to blinding of individuals delivering the intervention, excluding patients with missing outcome data (i.e., conducting per protocol analyses) or in relation to managing missing data. Future studies should use a more rigorous study design to ascertain the effectiveness of these interventions. Nevertheless, it is worth noting that blinding may not be suitable for all studies based on the nature of the interventions rendered.

### Characteristics of the Participants and Interventions

Participants across studies were similar in that they are functionally independent and were living in the community. Slightly more than half were single, divorced, widowed, or separated, and almost two-thirds lived alone. There was an overrepresentation of female participants (70.2%) in this review. This appears to be in line with Kosberg and Mangum’s (2002) sentiments that older men are less often portrayed in gerontological literature, and that there is insufficient research on the needs of older men. Age has been argued to be a “gendered phenomenon,” with gendered differences in individuals’ actions, sense-making, and social practices (Cislaghi & Heise, 2020). Recreational and educational groups have been argued to be “feminized’ (Russell, 2007, p. 181), for instance, users of recreational facilities for older adults are more likely females, those who were living alone and single (Bøen et al., 2010; Marhankova, 2014). Accordingly, the overrepresentation of women might be attributed to the gendered appeal of the activities. Men’s Sheds are male friendly organizations that were started in Australia to engage men in activities for health and well-being (Kelly et al., 2021), and a recent systematic review revealed their benefits on improving self-rated health, social isolation, and well-being among older men (Foettinger et al., 2022). This emphasizes that tailoring or designing interventions targeted at supporting older men might be necessary moving forward. Looking to future generations, older adults of the future are more likely to be technology savvy. Accordingly, shared activities such as computer or internet activities that they engage in may cross gender barriers that have been identified in previous studies involving the current or previous generations of older people.

Most studies delivered multicomponent interventions, which targeted more than one domain of healthy aging. Most supported lifelong learning, which refers to “personal or professional development, taken as a lifestyle of successful ageing … to compensate for some physical or cognitive deficiencies and participation, or simply to enjoy oneself” (Molina & Schettini, 2021, p. 111). Intervention delivery formats varied. Nearly two-thirds delivered interventions in group formats, although facilitating social support was not a focus in all. Accordingly, group interactions in these interventions could serve as a confounding variable. The duration of the interventions was widely ranging from 4 to 18 weeks. Interventions were delivered both by health and social care professionals, nonhealth professionals, or by volunteers. Nevertheless, intervention fidelity and/or training to deliver the intervention was not reported in most studies. Variations in intervention duration and attributes of those delivering interventions could have influenced results of the pooled analysis. Future studies can consider evaluating interventionist training and intervention fidelity. It may also be worth investigating the dose-response effect of interventions to identify optimal intervention parameters.

### Effects of Life Satisfaction and QoL

The meta-analysis revealed that non-exercise-based interventions had a large, but nonstatistically significant effect on life satisfaction. Likewise, the results from the narrative summary are mixed. On the contrary, both the pooled analysis and narrative summary indicate that non-exercise-based interventions led to statistically significant improvements in overall QoL. These findings correlate with findings from two systematic reviews and meta-analyses (Park et al., 2020; Vasodi et al., 2023) that found small but statistically significant effect size between physical activity and QoL. Likewise, this review revealed that the interventions had statistically significant effects on the mental health and psychological well-being QoL subscale. Comparing this with exercise-focused interventions, based on a meta-analysis of 32 studies, Arent et al. (2000) found that exercise had a moderate effect size on mood in healthy older adults. Likewise, a meta-analysis by Netz et al. (2005) had similar findings that exercise had a small but statistically significant effect on the psychological well-being of older adults. These findings suggest that both exercise and non-exercise-focused aging interventions could have similar effects on improving the overall QoL and QoL mental subscales in older adults. This has implications in that non-exercise-based interventions could be a viable option in supporting healthy aging particularly for older adults who are not able or motivated to engage in physical activities. Given that the effect sizes are small, it might be worth optimizing the interventions and carefully tailor them to participants’ context and needs to maximize their effects. For example, future trials should consider further optimizing interventions by grounding them based on relevant theories (e.g., behavioral change) and user-driven intervention development approaches (e.g., relevant codesign frameworks).

Results showed that non-exercise-based interventions led to very marginal improvements on improving the social health and functioning, role functioning (physical), and environmental subscales of QoL. According to Van Leeuwen et al. (2019), QoL subdomains often intertwine and influence each other. Influences in one QoL subdomain are also likely to influence other domains. It is not surprising that there were no or very marginal effects on improving perceived physical health and role functioning related to physical well-being, given that none of the studies focused on exercise as part of their interventions. Although most studies targeted lifelong learning and involved multicomponent interventions, variations in the characteristics of the interventions and intervention delivery as highlighted in the section above could have potentially influenced the effects or statistical significance in pooled analyses.

This work indicated the range of outcomes relevant to healthy aging programs. Quality of life is a highly relevant outcome capturing the impact of the interventions broadly for older people. Usefully, this nature of outcome may enable meaningful comparison of impacts and outcomes across intervention types, and future healthy aging trials, including both non-exercise and physical activity focused, should consider inclusion of the measurement of QOL outcomes.

### Strengths and Limitations

This is the first systematic review and meta-analysis of RCTs to investigate the effectiveness of non-exercise-based interventions in supporting healthy aging, particularly in relation to life satisfaction and QoL. It followed a rigorous process guided by PRISMA reporting guidelines. In addition, investigating intervention effects specifically relating to life satisfaction and QoL provided a common denominator for the synthesis and comparison of findings. Nevertheless, limitations need to be acknowledged. Although participants should be a rather homogenous group (i.e., functionally independent older adults living in the community), effects of a range of non-exercise-based interventions were synthesized. The number of participants in some studies were small and therefore are underpowered to detect significant effects. This, along with intervention heterogeneity, might have influenced the results of the pooled analysis. Although subgroup analysis was conducted when possible, subgroup analysis based on the intervention types was limited. Future trials should be carefully planned and powered, be reported, and conducted following relevant guidelines like CONSORT (Consolidated Standards of Reporting Trials). Next, the risk of bias is moderate to high in most studies. This could be attributed to the pragmatic nature of the studies, where many studies were not able to sufficiently perform blinding, and that results were primarily based on patient-reported outcome measures. Studies in other languages were excluded, and trial registry records and conference abstracts have not been searched. This may have limited the comprehensiveness of this review. Studies that did not present sufficient raw data (mean, *SD*) or studies that presented data in an inappropriate format were excluded from the meta-analysis. Finally, although this study focused on healthy older populations, it is important to note that many people experience some form of disability, have medical diagnoses or comorbidities. Correspondingly, as these study findings might be used to support the development or further evaluation of interventions, intervention developers should be mindful of being inclusive to older adults regardless of their medical comorbidities.

## Conclusion

This systematic review and meta-analysis synthesized evidence on the effectiveness of non-exercise-based interventions on functionally independent, community-dwelling older adults, based on 35 RCTs. Interventions mostly involved multiple components, and the majority were targeted at supporting lifelong learning. Moderate or high risks of bias were demonstrated in most studies, particularly in relation to blinding and the management and/or lack of analysis of missing outcome data. Pooled results indicate that non-exercise-based interventions have positive, statistically significant effects on the overall QoL in older adults, as well as in mental health, social health, and environment QoL subscales. The effect sizes are small, and future studies can consider optimizing interventions and their potential effects through contextually relevant and targeted approaches such as accounting for gendered considerations.

## Supplementary Material

gnae156_suppl_Supplementary_Materials

## Data Availability

Data sharing is not applicable to this article as this is a secondary study. No primary datasets were generated or analysed during the current study. The search terms and strategies are provided to allow for replication. Supporting data and materials used in this paper can be accessed online through academic databases, and the datasets used and/or analysed during this study are available from the corresponding author upon reasonable request. We did not access whether the studies reported in our review were preregistered.
