# Supplementary material for: Non–Exercise-Based Interventions to Support Healthy Aging in Older Adults: A Systematic Review and Meta-Analysis of Randomized Controlled Trials
Source: Gerontologist. 2024 Oct 30;65(2):gnae156. doi: 10.1093/geront/gnae156 (PMC11979768; doi:10.1093/geront/gnae156)
Supplement: gnae156_suppl_Supplementary_Materials [file gnae156_suppl_supplementary_materials.docx]

**Supplementary Material**

**Appendix 1: Sample Search Strategy**

**CINAHL (via EBSCO)**

S1: Healthy ag*ing OR successful ag*ing OR “positive ag*ing OR ag*ing well OR active ag*ing

S2: diet OR nutrition

S3: self*awareness OR self*knowledge OR insight

S4: attitude OR behavio*r OR self*efficacy OR confidence

S5: social support OR social network OR social participation OR social engagement OR social environment OR social support OR social relations*

S6: finance* OR financ* OR money OR incentive

S7: community participation OR community engagement OR community involvement OR civic participation OR civic engagement

S8: independent living OR activities of daily living OR instrumental activities OR independence OR self care

S9: physical environment OR built environment

S10: health service* OR social service* OR ( health and social service* ) OR health facilit* OR social facilit* OR ( health and social facilit* )

S11: fall prevention OR prevent fall* OR reduce fall* OR fall reduc*

S12: cognition OR cognitive

S13: self manage* OR medication manage* OR medication adherence

S14: social health

S15: sleep

S16: quality of life OR satisfaction OR well*being

S17: randomised controlled trial OR randomized controlled trial OR randomised trial OR randomized trial OR controlled trial OR randomised controlled study OR randomized controlled study

S18: community dwelling OR community-dwelling OR living at home OR live at home OR living in the community OR living independently

S19: older adult* OR elderly OR aged OR older people OR senior* OR older population OR old age

S20: S1 OR S2 OR S3 OR S4 OR S5 OR S6 OR S7 OR S8 OR S9 OR S10 OR S11 OR S12 OR S13 OR S14 OR S15 OR S16

S21: S17 AND S18 AND S19 AND S20

**Appendix 2: Inclusion/exclusion criteria (PICOS)**

|  | **Inclusion criteria** | **Exclusion criteria** |
| --- | --- | --- |
| **Population** | - Healthy older adults (aged 55 and above), i.e.,   - Independent in self-care   - No explicit health conditions  (i.e., no reported cognitive impairment, physical impairment, major psychiatric disorders or neurological diseases) limiting independence in self-care - At risk of acquiring cognitive or physical impairment (e.g., risk of depression, prodromal depression, pre-frailty) - Living in the community | - Older adults aged <55 years old - Existing cognitive impairment (e.g., mild cognitive impairment, early dementia) - Existing physical impairment or neurological diseases (e.g., stroke, Parkinson’s disease, frailty) - Unable to be independent or requires assistance in self-care activities - Living in residential care services, hospital (acute/respite) admission |
| **Intervention** | Non-physical-focused interventions targeted at **supporting older adults** to live healthily within the community. These include interventions targeted at:   - Self-management (e.g., medication/dietary self-management) - Self-awareness, - Attitude and behaviours - Sleep - Learning - Social support, social environment and community engagement - Independence in daily living - Financial security - Physical environment - Health and social services | - Targeted at **non-older adults** (e.g., family members, community, care professionals, care providers) - Physical-based interventions, whether as a standalone or combined intervention to isolate the effects of exercise on outcomes (e.g., strengthening and balance training, exergaming, Taichi) mindfulness training and exercise) - Fall prevention and risk reduction - Prevent hospitalisation (e.g., older adults recently discharged from the hospital) - Chronic disease management (e.g., diabetes self-management) |
| **Comparator** | - No intervention (treatment as usual), waiting list or minimal intervention | - Control group involves delivery of another intervention |
| **Outcomes** | Primary outcomes:   - Quality of life - Satisfaction and/or purpose in life | - No outcomes pertaining to quality of life or life satisfaction - Other outcomes   Such as preventing re-admission into hospital, preventing nursing home/ admission into residential care) |
| **Study design** | - From inception - Randomised controlled trial published in peer-reviewed journals | - Non-randomised controlled trials (e.g., quasi-experimental/feasibility studies) - Non-interventional studies (e.g., correlational studies, editorial) - Grey literature (e.g., conference proceedings, editorials) |

**Appendix 3. Summary of study characteristics**

| **Author, year** | **Country** | **Age: T/C mean (SD/range)** | **Sample size:**  **Total (T/C)** | **Intervention** | **Control** | **Intervention facilitator / deliverer** | **Intervention duration** |
| --- | --- | --- | --- | --- | --- | --- | --- |
| Arola, 2020 | Sweden | 74.0 (3.4)/  74.2 (3.4) | 131 (56/75) | Group meetings, comprising visits from inter-disciplinary team (reflections about general challenges related to ageing and health in daily life) | Treatment as usual (access to healthcare services) | 1 occupational therapist, 1 physiotherapist, 1 registered nurse, and/or 1 social worker | 4 weeks  (1.5-2h weekly) |
| Barnett, 2022 | USA | 72.8 (10.2)/ 72.7 (8.0) | 292  (150/142) | Healthy eating for successful living in older adults, aimed at increasing knowledge of healthy eating habits and identify food choices for overall health | Treatment as usual | Program leaders  who were trained & certified; dietician/ nutritionist to answer questions | 6 weeks  (2.5h weekly) |
| Beauchet, 2021 | Canada | 71.5 (4.7) / 71.9 (5.0) | 165  (82/83) | Participatory art-based activity consisting of group-based art creation at a museum, involving a range of arts (e.g., painting, stained glass painting) | Treatment as usual | Arts and culture facilitators | 12 weeks  (2h weekly) |
| Beauchet, 2022a | Japan, Canada | 70.7 (6.1)/  71.6 (4.6) | 228  (119/109) | Participatory art-based activity consisting of group-based art creation at a museum, involving a range of arts (e.g., painting, stained glass painting). Activities were specific to each country | Treatment as usual | Arts and culture facilitators | 12 weeks  (2h weekly) |
| Beauchet, 2022b | Canada | 75.0 (4.6)/  74.3 (5.1) | 106  (53/53) | Virtual (Zoom) guided museum tours, which involved: presenting visit objectives, dialogic style tour with museum guides & discussions | Treatment as usual | Trained museum guides | 3 months  (45 mins weekly) |
| Blancafort, 2021 | Spain | 73.4 (6.9)/  73.9 (7.0) | 360  (194/164) | Multicomponent community program targeting a range of outcomes and new skills and behaviours, involving local outings to public spaces (e.g., supermarket, market) | Waiting list | Trained health &social care professionals (5 nurses, 2 social workers, 2 general practitioners | 12 weeks  (2h weekly) |
| Clark, 1997; Clark, 2001 | USA | - | 361  (122/239) | Support older adults to incorporate positive changes in their ongoing lifestyles (e.g., health relevant behaviours, social relationships) through didactic presentations, peer change, personal experiences exploration | Social activities e.g., crafts or no activities (treatment as usual) | Occupational therapists  (40h of training to standardise intervention) | 9 months (2h weekly group sessions & 9h one-to-one sessions) |
| Clark, 2012 | USA | 74.81 (7.8)/  74.90 (7.6) | 460  (232/228) | Support older adults to better appreciate the importance of meaningful activity in their lives, impart specific knowledge about how to select or perform activities to achieve a healthy and satisfying lifestyle through didactic teaching and direct experience with a broad range of activities | Social activities e.g., crafts or no activities (treatment as usual) | Occupational therapists who were trained to deliver intervention as per protocol | 6 months  (2h weekly group sessions & up to 10h of one-to-one session) |
| Czaja, 2018 | USA | 75.3 (7.4)/  76.9 (7.3) | 300 (150/150) | Providing a Personal Reminder Information and Social Management System (PRISM) to support older adults, providing internet access, annotated resource guide (e.g., information on relevant national/local organisations), tab to connect with other PRISM users. Initial training provided; technical support available | Notebook with printed content similar to that within PRISM | No information on who provided the initial training and provided technical support | 12 months |
| Godwin, 2016 | Canada | 85.3 (4.5)/  71.8 (7.0) | 236  (121/115) | Home based - Initial evaluation of ADLs, disability, medication compliance and use, safety issues, home and personal hygiene, understanding of medical/health issues. Individualised plans were developed, participants were supported to meet goals, optimise resource use | Treatment as usual | Primary care nurse specialist | 1 year  (1-2h for initial assessment, & 8 subsequent follow-up visits) |
| Hayashi, 2016 | Japan | 70.4 (4.1)/  68.7 (8.4) | 73  (34/39) | Arts intervention modified for cultural specificity (Japan). This included art making workshops (interactive, hands-on activities for creativity, fine motor skills, handicraft techniques and observation skills), dialogue-based art appreciation programs and group discussions | Treatment as usual | Professional artists and Arts Alive facilitators | 12 weeks  (12 workshops) + 30-mins monthly arts appreciation |
| Ho, 2021 | Singapore | 72.6 (7.56)/ 73.6 (5.5) | 34  (17/17) | ARTISAN intervention, involving weekly themes, schedules, art activities jointly developed by community representatives, artists and the research team – Group based intergenerational arts and heritage intervention which included  guided museum tours, professionally led artmaking, guided storytelling, reflective writing | Waiting list | A docent, a professional artist or trained art therapist, and research staff | 5 weeks  (weekly, total 15h) |
| Kazazi, 2021 | Iran | 65.42 (5.4)/  64.38 (5.0) | 52  (26/26) | Computer based cognitive training intervention aimed at improving working memory and selective attention. The program consists of 10 graded progressive tasks, comprising 10 levels; moving to a higher-level upon scoring 80% | Educational workshops age related cognitive changes & disorders | No information | 10 weeks  (45 mins twice weekly), 1 pre- and 1 post-evaluation |
| Keisari, 2022 | Israel | 78.65 (6.91)/  80.60 (6.81) | 78  (40/38) | Integrative intervention involving participation in playback theatre, based on life review done in groups. Participants share one or several life cross-road stories, and co-create a theatrical improvision with therapist | Treatment as usual | Drama therapist and social worker | 12 weeks (90mins, weekly) |
| Ko, 2011 | Korea | 76.33 (6.44)/ 73.92 (7.79) | 109  (48/61) | Laughter therapy, where moderator explained effects of laughter and educated them about laughter therapy. Four meetings took place in total, where participants (in groups) were led into singing, dancing activities, positive thinking, repeating positive words and laughter mediation | Treatment as usual | Nurse (certified in laughter therapy by the Korean Laughter Therapy Professional Association) | 4 weeks  (1h weekly) |
| Ko, 2016 | Korea | 71.07 (5.51)/  72.65 (5.73) | 94  (46/48) | Insect assisted therapy (crickets) – Individuals educated about intervention, how to care for the insects & making healthy lifestyle choices; weekly phone calls to encourage care of the crickets and adhere to healthy lifestyle choices | Two lectures (1h each) on healthy lifestyle choices | No information | 8 weeks |
| Kwok, 2013 | Hong Kong | - | 176  (86/90) | Cognitive training with specific elements and modifications tailored to local context for cultural relevance. Modules include imagery memory training, community-understanding. Education was also provided (e.g., memory deterioration, attention training, environmental awareness, lifestyle redesign) | Treatment as usual | Facilitation provided during groups, no information regarding facilitators | 8 weeks  (1h weekly) |
| Kwon, 2015 | Korea | 77.41 (6.75) /  73.50 (7.35) | 93  (43/46) | Wellness counselling involving four steps: 1) introduction of the Wheel of Wellness model; 2) assessing wellness issues, 3) intentional intervention, including setting a personal wellness goal & plan to enhance wellness in selected subtasks, 4) evaluation, follow-up, support | Treatment as usual | Nurses with a master’s degree or a doctoral degree | 4 weeks  (1h weekly) |
| Lai, 2019 | Hong Kong | 77.98 (7.15)/ 76.20 (7.65) | 244  (124/120) | Lifestyle book creation – participants facilitated to provide an account of past events significant to their life history (e.g., talking about their life, validating written content). Several cycles of meetings and interviews were conducted, pictures and memorabilia were used to facilitate sharing. The end product was a life storybook | Social activities unrelated to life story work | Volunteers facilitated group sessions | 4-6 weeks  (30-60mins weekly) |
| Lee, 2010 | Korea | 75.5 (7.1)/ 77.7 (6.5) | 66  (31/35) | Music intervention, each played for 30 minutes. Musical pieces were loaded onto an MP3 player to allow participants to choose their preferred music. MP3 player use was demonstrated, music selection introduced, and music intervention was conducted (quiet room) | Treatment as usual | Unfacilitated (researchers provided support to set up) | 4 weeks  (30min weekly) |
| Liu, 2023 | Taiwan | 72.90 (4.45)/ 72.78 (4.52) | 100  (50/50) | Remote bidirectional interaction, where online group interventions were facilitated, and live classes (static and dynamic content). Content included instructions on smartphone use, stretching). The content was designed by an assistant professor of occupational therapy, a licensed long-term care program instructor & was reviewed by an assistant professor of nursing | Unidirectional remote interaction (e.g., YouTube videos) | Long-term care program instructor | 12 weeks  (1h daily from Monday to Friday, 60h total) |
| McCarthy, 2018 | USA | - | 20  (10/10) | Psychoeducation in groups, with sessions focusing on self-transcendence related topics (e.g., mindfulness practice, creative processes such as art projects, writing, deep breathing) | Waitlist control | A gerontological nurse & a research associate (and consultation with a social worker) | 8 weeks  (1.5h weekly) |
| Mountain, 2014 | UK | 81.8 (5.8)/ 80.1 (3.7) | 70  (35/35) | Increase extent of their social networks through the use of one-to-one telephone befriending. Volunteers were trained and made 10-20 min calls once a week for up to 6 weeks with allocated participants. This was followed by group telephone befriending for 6 weeks after | Treatment as usual | Volunteers who received training from a customer engagement manger (1-2.5h per session for 2-7 sessions) | 18 weeks:  6 weeks of one-to-one sessions, then 12 weeks of group sessions |
| Mountain, 2017 | UK | 72.9 (65-92)/  71.3 (65-90) | 288  (145/143) | Manualised intervention using Lifestyle matters – designed to assist participants to improve wellbeing and avoid decline with social isolation and poor mental health. The emphasis was on identifying participant goals and empowerment | Treatment as usual | National Health Service (NHS) or  social care staff who were trained and supervised by occupational therapists | 4 months (weekly) |
| Rantanen, 2020 | Finland | - | 204  (101/103) | Individual counselling where participants received an information booklet about active ageing, followed by 4 phone counselling sessions to provide additional support, feedback and encouragement, with an aim to support autonomous motivation and foresee the benefits of increased of new activities | Printed general health materials (e.g., exercise, nutrition) | Counseller | 1 year  (One face-to-face session & 4 phone-based sessions) |
| Ristolainen, 2020 | Finland | 76.8 (7.2) | 392 (185/207) | Group meetings (participatory group-based care management) focusing on providing social support, counselling, and activities | Treatment as usual | A care manager and a researcher | 6 months  (5 sessions, 2-3h per session) |
| Saito, 2012 | Japan | 72.6 (4.4)/ 72.8 (4.8) | 60  (20/40) | Group-based educational, cognitive, and social support program aimed at preventing social isolation by improving community knowledge, networking with other participants and various community gatekeepers. | Waitlist control group | Staff who were participating in the  program (no further information provided) | 8 weeks  (2h x 4 sessions conducted fortnightly) |
| Shorey, 2021 | Singapore | 71.1 (5.9)/  67.7 (7.4) | 28  (14/14) | Integrated solution-focused brief therapy (SFBT) involving psychoeducation and structured life review and mindfulness-based training developed by experts. The SFBT focuses on strengths and solutions rather than problems and deficits; delivered through group sessions | Treatment as usual | A volunteer who was trained by the principal, and a mindfulness practitioner | 7 sessions (duration not stated) |
| Sledgers, 2008 | The Netherlands | 64-75 | 191 (62/129) | Computer training (plenary discussions of computer and internet topics, operating a computer & internet applications) and computer intervention involving use of a personal computer based on needs | Treatment as usual or computer training | Computer instructors and helpdesk support | Unclear (at least 4.5 months) |
| Tan, 2021 | Singapore | 74.9 (5.7)/  75.8 (6.2) | 64  (32/32) | Resource enhancement and activation program (REAP), which is aimed at promoting understanding of external life challenges confronting older people, understanding personal beliefs, thoughts, and emotions. Participants can also review relevant external resources | Treatment as usual | Facilitated by the researcher and a trained assistant | 12 weeks  (24 activities) |
| Tamasco, 2022 | Chile | 70.8 (6.5)/  67.8 (8.5) | 69  (37/32) | Initial visit evaluated by a medical/health professional (doctor, physiotherapist/ psychologist) before using the “Quida system”, a non-intrusive integrated monitoring system to monitor the daily activities of older adults, detect and alert family or contact of risk events e.g., gas leak, fall, emergency button activation | Treatment as usual | Non-facilitated | 1 month |
| Wang, 2023 | Taiwan | 71.9 (6.5)/  72.1 (7.6) | 38  (19/19) | Health education on mental health at the outset. A magic instructor led the subsequent program to teach magic tricks, involving demonstration, hands-on practice and participants’ presentation of their learnt magic skills | Health education on mental health | An experienced instructor (was both a family physician and a professional magician) | 6 weeks  (Twice weekly, 90mins per session) |
| Wilhemson, 2013 | Sweden | Intervention 1:  86 (80-97)/  86 (80-94)  Intervention 2:  85 (80-94) | 459  (174, 171/ 114) | Intervention 1: Preventative home visit involving a home visit made by an interdisciplinary team staff, to render verbal and written advice on local services resources, falls risks - OR  Intervention 2: Group meeting (discuss about the ageing process, tools & problem-solving strategies) and a follow-up visit | Treatment as usual | Inter-disciplinary team: 1 occupational therapist, 1 physiotherapist, 1 registered nurse, and/or 1 social worker | Four weekly meetings + one follow-up home visit 2-3 weeks after |
| Wong, 2020 | Hong Kong | 78.0 (8.36)/ 78.0 (7.47) | 457 (230/227) | Aimed at supporting self-care by assessing, promoting mental and physical health. This was done through an initial home visit and subsequent weekly telephone calls (3 weeks). Thereafter, biweekly home visits or telephone phone follow up were conducted for 2 months. | Treatment as usual | Registered nurse, supported by community workers and social workers (telephone communication skills training) | 3 months |
| Yamada, 2010 | Japan | 72.6 (4.3)/ 72.3 (4.7) | 63  (30/33) | Model of Human Occupation (MOHO) based program to provide opportunities for participants to reflect on their occupational lives using concepts from MOHO, discuss and enact ways in which they could better meet occupational needs. Each session included a lecture followed by group discussions. | Craft activities and informal conversations during the activities | Occupational therapists | 8 months  (2h twice monthly, 15 sessions in total) |

**Appendix 4. Healthy ageing domains summary**

| **Author, year** |  | **Healthy ageing domains** | | | | | | | | | | | |
| --- | --- | --- | --- | --- | --- | --- | --- | --- | --- | --- | --- | --- | --- |
|  | **Physical** |  | **Mental/cognitive** | | | | | **Social** | | | | **Overlap** |  |
|  | *Diet* | *Self-awareness* | | *Attitude* | *Lifelong learning* | *Faith* | *Social support* | | *Financial security* | *Community engagement* | *Independence* | | |
| Arola, 2020 |  | ✔ | |  | ✔ |  |  | |  |  |  | | |
| Barnett, 2022 | ✔ | ✔ | |  |  |  |  | |  |  |  | | |
| Beauchet, 2021 |  |  | |  | ✔ |  |  | |  | ✔ |  | | |
| Beauchet, 2022a |  |  | |  | ✔ |  |  | |  | ✔ |  | | |
| Beauchet, 2022b |  |  | |  |  |  | ✔ | |  | ✔ |  | | |
| Blancafort, 2021 |  |  | |  | ✔ |  | ✔ | |  | ✔ | ✔ | | |
| Clark, 1997, 2001, 2012 |  | ✔ | |  | ✔ |  |  | |  |  | ✔ | | |
| Czaja, 2018 |  |  | |  |  |  | ✔ | |  | ✔ |  | | |
| Godwin, 2016 |  |  | |  | ✔ |  | ✔ | |  |  |  | | |
| Hayashi, 2016 |  |  | |  | ✔ |  |  | |  | ✔ |  | | |
| Ho, 2021 |  |  | |  | ✔ |  | ✔ | |  | ✔ |  | | |
| Kazazi, 2021 |  |  | |  | ✔* |  |  | |  |  |  | | |
| Keisari, 2022 |  |  | | ✔ | ✔ |  | ✔ | |  |  |  | | |
| Ko, 2011 |  |  | |  | ✔ |  |  | |  |  |  | | |
| Ko, 2016 |  |  | |  |  |  |  | |  |  |  | | |
| Kwok, 2013 |  |  | |  | ✔ |  |  | |  |  |  | | |
| Kwon, 2015 |  | ✔ | |  | ✔ |  |  | |  |  | ✔ | | |
| Lai, 2019 |  | ✔ | |  | ✔ |  |  | |  | ✔ |  | | |
| Lee, 2010 |  |  | |  | ✔ |  |  | |  |  |  | | |
| Liu, 2023 |  |  | |  | ✔ |  | ✔ | |  |  |  | | |
| McCarthy, 2018 |  | ✔ | | ✔ | ✔ |  | ✔ | |  |  |  | | |
| Mountain, 2014 |  |  | |  |  |  | ✔ | |  |  |  | | |
| Mountain, 2017 |  |  | | ✔ | ✔ |  |  | |  | ✔ | ✔ | | |
| Rantanen, 2020 |  |  | |  | ✔ |  | ✔ | |  | ✔ |  | | |
| Ristolainen, 2020 |  |  | |  | ✔ |  | ✔ | |  | ✔ |  | | |
| Saito, 2012 |  |  | |  |  |  | ✔ | |  | ✔ |  | | |
| Shorey, 2021 |  |  | | ✔ | ✔ |  | ✔ | |  | ✔ |  | | |
| Tan, 2021 |  | ✔ | |  | ✔ |  |  | |  | ✔ |  | | |
| Tamasco, 2022 |  |  | |  |  |  |  | |  |  | ✔ | | |
| Wang, 2023 |  |  | |  | ✔ |  |  | |  |  |  | | |
| Wilhemson, 2013 |  |  | |  | ✔ |  | ✔ | |  |  | ✔ | | |
| Wong, 2020 |  |  | |  |  |  |  | |  | ✔ |  | | |
| Yamada, 2010 |  |  | |  |  |  |  | |  |  | ✔ | | |

**Appendix 5: Forest plots of QoL subcomponents**


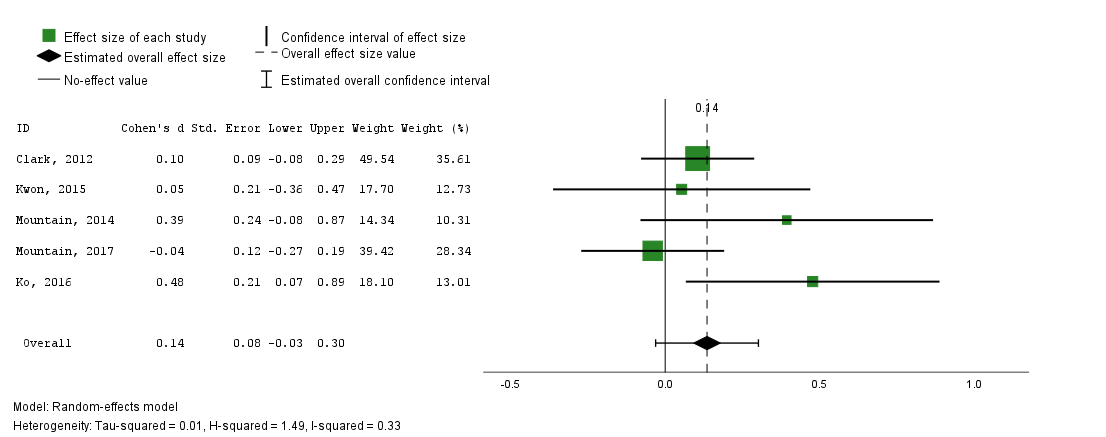
**Supplementary Figure 1: Forest plot –Intervention effects (mental component summary)**

*(Alt text): Forest plot of effects on mental component*

**Supplementary**
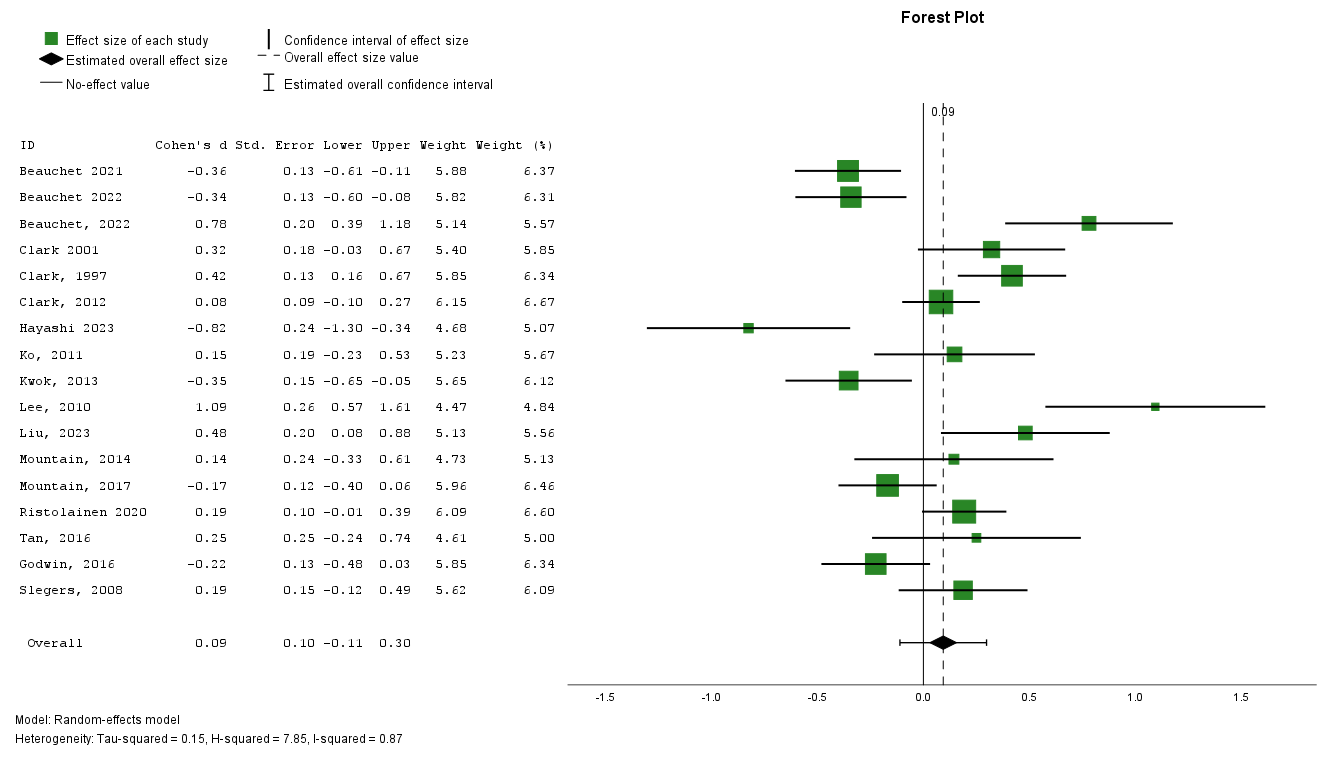
**Figure 2: Forest plot –Intervention effects (physical health & function)**

*(Alt text): Forest plot of effects on physical health and function*


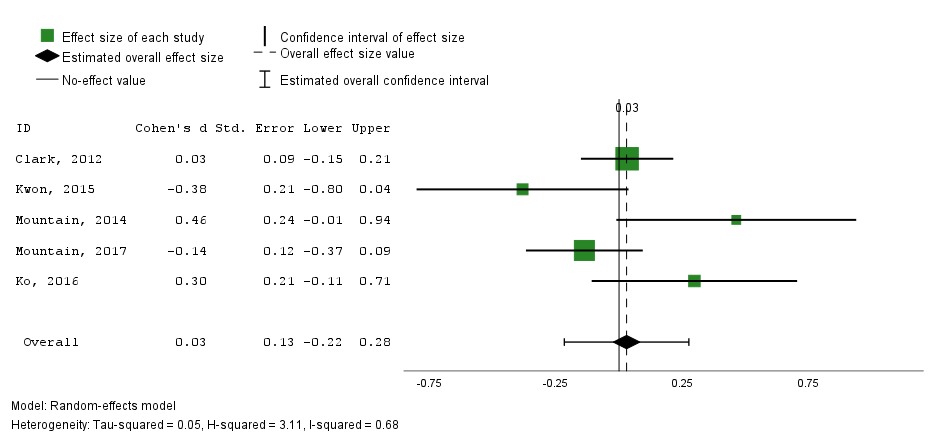

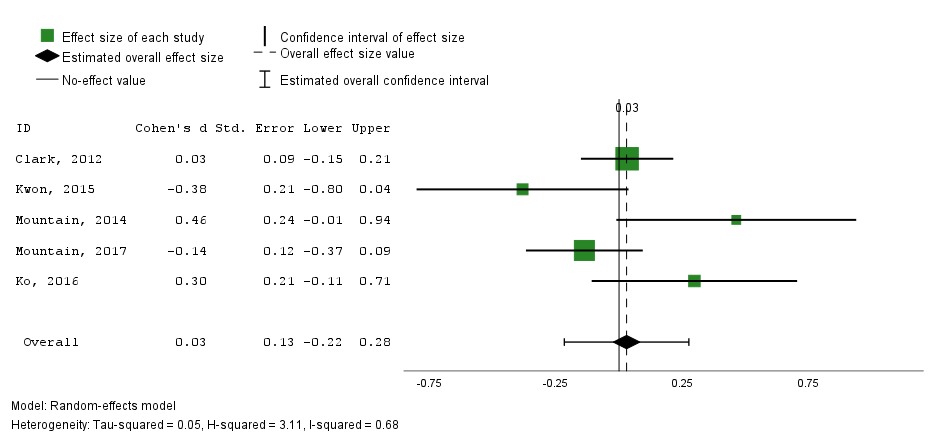
 **Supplementary Figure 3: Forest plot –Intervention effects (physical component summary)**

*(Alt text): Forest plot of effects on physical component*


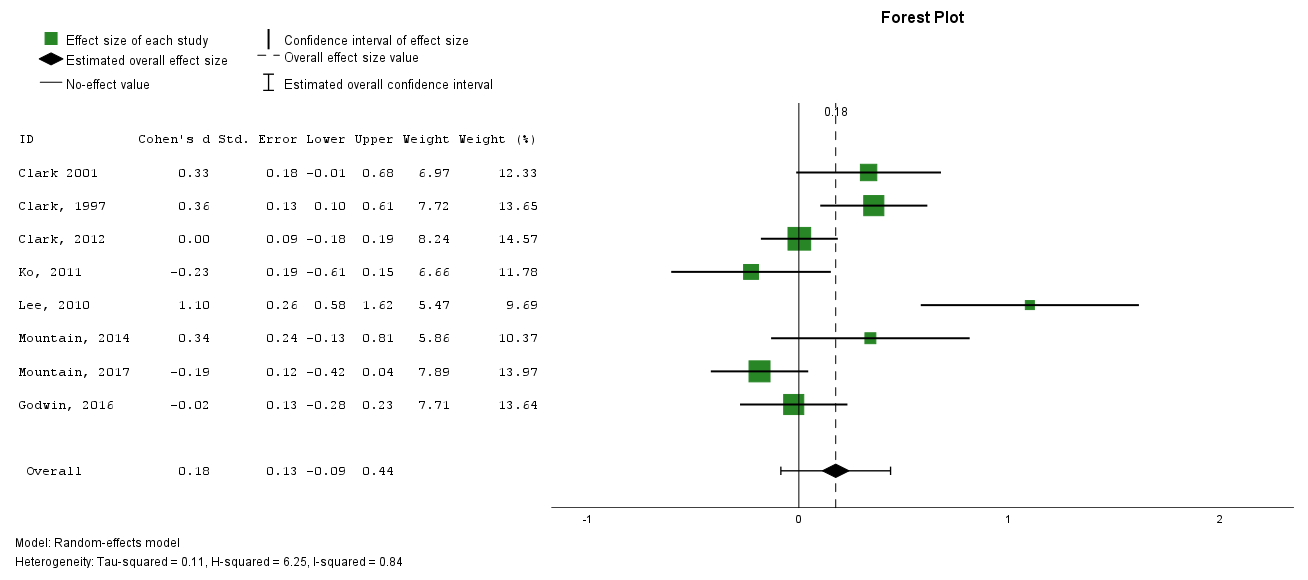
**Supplementary Figure 4: Forest plot –Intervention effects (Role functioning, emotional)**

*(Alt text): Forest plot of effects on role functioning - emotional*

**Appendix 6. PRISMA Checklist**

**PRISMA Checklist**

| **Section and Topic** | **Item #** | **Checklist item** | **Location where item is reported** |
| --- | --- | --- | --- |
| **TITLE** | | |  |
| Title | 1 | Identify the report as a systematic review. | Title page |
| **ABSTRACT** | | |  |
| Abstract | 2 | See the PRISMA 2020 for Abstracts checklist. | p2 |
| **INTRODUCTION** | | |  |
| Rationale | 3 | Describe the rationale for the review in the context of existing knowledge. | p3-4 |
| Objectives | 4 | Provide an explicit statement of the objective(s) or question(s) the review addresses. | p4 |
| **METHODS** | | |  |
| Eligibility criteria | 5 | Specify the inclusion and exclusion criteria for the review and how studies were grouped for the syntheses. | p5-6, Appendix 1 |
| Information sources | 6 | Specify all databases, registers, websites, organisations, reference lists and other sources searched or consulted to identify studies. Specify the date when each source was last searched or consulted. | p4 |
| Search strategy | 7 | Present the full search strategies for all databases, registers and websites, including any filters and limits used. | p5 |
| Selection process | 8 | Specify the methods used to decide whether a study met the inclusion criteria of the review, including how many reviewers screened each record and each report retrieved, whether they worked independently, and if applicable, details of automation tools used in the process. | p6 |
| Data collection process | 9 | Specify the methods used to collect data from reports, including how many reviewers collected data from each report, whether they worked independently, any processes for obtaining or confirming data from study investigators, and if applicable, details of automation tools used in the process. | p6 |
| Data items | 10a | List and define all outcomes for which data were sought. Specify whether all results that were compatible with each outcome domain in each study were sought (e.g. for all measures, time points, analyses), and if not, the methods used to decide which results to collect. | p6 |
|  | 10b | List and define all other variables for which data were sought (e.g. participant and intervention characteristics, funding sources). Describe any assumptions made about any missing or unclear information. | p6 |
| Study risk of bias assessment | 11 | Specify the methods used to assess risk of bias in the included studies, including details of the tool(s) used, how many reviewers assessed each study and whether they worked independently, and if applicable, details of automation tools used in the process. | p6 |
| Effect measures | 12 | Specify for each outcome the effect measure(s) (e.g. risk ratio, mean difference) used in the synthesis or presentation of results. | p7 |
| Synthesis methods | 13a | Describe the processes used to decide which studies were eligible for each synthesis (e.g. tabulating the study intervention characteristics and comparing against the planned groups for each synthesis (item #5)). | p7 |
|  | 13b | Describe any methods required to prepare the data for presentation or synthesis, such as handling of missing summary statistics, or data conversions. | p7 |
|  | 13c | Describe any methods used to tabulate or visually display results of individual studies and syntheses. | p7 |
|  | 13d | Describe any methods used to synthesize results and provide a rationale for the choice(s). If meta-analysis was performed, describe the model(s), method(s) to identify the presence and extent of statistical heterogeneity, and software package(s) used. | p7 |
|  | 13e | Describe any methods used to explore possible causes of heterogeneity among study results (e.g. subgroup analysis, meta-regression). | p7 |
|  | 13f | Describe any sensitivity analyses conducted to assess robustness of the synthesized results. |  |
| Reporting bias assessment | 14 | Describe any methods used to assess risk of bias due to missing results in a synthesis (arising from reporting biases). | p7 |
| Certainty assessment | 15 | Describe any methods used to assess certainty (or confidence) in the body of evidence for an outcome. | p7 |
| **RESULTS** | | |  |
| Study selection | 16a | Describe the results of the search and selection process, from the number of records identified in the search to the number of studies included in the review, ideally using a flow diagram. | Figure 1 |
|  | 16b | Cite studies that might appear to meet the inclusion criteria, but which were excluded, and explain why they were excluded. | Figure 1 |
| Study characteristics | 17 | Cite each included study and present its characteristics. | Appendix 2 |
| Risk of bias in studies | 18 | Present assessments of risk of bias for each included study. | p8-10 |
| Results of individual studies | 19 | For all outcomes, present, for each study: (a) summary statistics for each group (where appropriate) and (b) an effect estimate and its precision (e.g. confidence/credible interval), ideally using structured tables or plots. | Appendix 4,  Figures 3-9,  p10-13 |
| Results of syntheses | 20a | For each synthesis, briefly summarise the characteristics and risk of bias among contributing studies. | Appendix 4,  Figures 3-9,  p10-13 |
|  | 20b | Present results of all statistical syntheses conducted. If meta-analysis was done, present for each the summary estimate and its precision (e.g. confidence/credible interval) and measures of statistical heterogeneity. If comparing groups, describe the direction of the effect. | Appendix 4,  Figures 3-9,  p10-13 |
|  | 20c | Present results of all investigations of possible causes of heterogeneity among study results. | p8-10 |
|  | 20d | Present results of all sensitivity analyses conducted to assess the robustness of the synthesized results. |  |
| Reporting biases | 21 | Present assessments of risk of bias due to missing results (arising from reporting biases) for each synthesis assessed. | p7 |
| Certainty of evidence | 22 | Present assessments of certainty (or confidence) in the body of evidence for each outcome assessed. | Appendix 4,  Figures 3-9,  p10-13 |
| **DISCUSSION** | | |  |
| Discussion | 23a | Provide a general interpretation of the results in the context of other evidence. | p13-16 |
|  | 23b | Discuss any limitations of the evidence included in the review. | p16-17 |
|  | 23c | Discuss any limitations of the review processes used. | p16-17 |
|  | 23d | Discuss implications of the results for practice, policy, and future research. | p10-17 |
| **OTHER INFORMATION** | | |  |
| Registration and protocol | 24a | Provide registration information for the review, including register name and registration number, or state that the review was not registered. | p4 |
|  | 24b | Indicate where the review protocol can be accessed, or state that a protocol was not prepared. | p4 |
|  | 24c | Describe and explain any amendments to information provided at registration or in the protocol. | p5 |
| Support | 25 | Describe sources of financial or non-financial support for the review, and the role of the funders or sponsors in the review. | p18 |
| Competing interests | 26 | Declare any competing interests of review authors. | P18 |
| Availability of data, code and other materials | 27 | Report which of the following are publicly available and where they can be found: template data collection forms; data extracted from included studies; data used for all analyses; analytic code; any other materials used in the review. | n/a |

*From:*  Page MJ, McKenzie JE, Bossuyt PM, Boutron I, Hoffmann TC, Mulrow CD, et al. The PRISMA 2020 statement: an updated guideline for reporting systematic reviews. BMJ 2021;372:n71. doi: 10.1136/bmj.n71
